# Radiation and Checkpoint Inhibitor Immunotherapy Lead to Long Term Disease Control in a Metastatic RCC patient With Brain Metastases

**DOI:** 10.3389/fonc.2020.566070

**Published:** 2020-09-23

**Authors:** Maria Levitin, Joel Ofori, Woo Jae Shin, Jiayi Huang, Mackenzie Daly, Dengfeng Cao, Russell Pachynski

**Affiliations:** ^1^Barbara Ann Karmanos Cancer Institute, Detroit, MI, United States; ^2^Washington University School of Medicine, St. Louis, MO, United States; ^3^Division of Oncology, Department of Medicine, Washington University School of Medicine, St. Louis, MO, United States; ^4^Department of Radiation Oncology, Washington University School of Medicine, St. Louis, MO, United States; ^5^Department of Pathology and Immunology, Washington University School of Medicine, St. Louis, MO, United States; ^6^Bursky Center for Human Immunology and Immunotherapy Programs (CHiiPs), Washington University School of Medicine, St. Louis, MO, United States

**Keywords:** renal cell carcinoma (RCC), brain metastases (BMs), immunotherapy, SBRT (stereotactic body radiation therapy), abscopal

## Abstract

Renal cell carcinoma (RCC) comprises 4.2% of all new cancer cases in the United States and 30% of cases are metastatic (mRCC) at diagnosis. Brain metastatic RCC historically has poor prognosis, but the development of immune checkpoint inhibitors has revolutionized their care and may be successfully combined with SBRT to improve prognosis. Here, we present a case of a patient with mRCC who had brain metastases treated with concurrent immune checkpoint inhibitors and SBRT. He continues to survive with good functional status years following his initial diagnosis. We discuss the relevant history regarding treatment approach in patients with brain metastatic RCC, ongoing trials focusing on the combination of immunotherapy and radiation, and the potential and promise of the abscopal effect.

## Introduction

Renal cell carcinoma (RCC) comprises 4.2% of all new cancer cases in the United States, with an estimated 73,820 diagnoses in 2019 (1, 2). Thirty percent of cases are metastatic at the time of diagnosis (3) and the incidence of brain metastasis over the course of 5 years is 9.8% (4). Prognosis without treatment is poor, with median survival of about 3 to 4 months (5). Prior to the use of targeted agents, mRCC was treated with interferon and the possible addition of surgery, whole brain radiotherapy, or radiosurgery for brain metastases. The prognosis for patients with brain metastases stagnated at around 3–7 months (5, 6). The development of targeted agents represented the first appreciable increase in life expectancy to a median overall survival (OS) of 9.2 months (7, 8). The management of mRCC has been revolutionized with the development of immune checkpoint inhibitors. Nivolumab is an immunoglobulin (Ig) G4 antibody against human programmed death (PD) 1 that blocks the interaction between PD-1 and its ligands PD-L1 and PD-L2. This helps limit the downregulation of immunostimulatory cytokines and exhaustion that occur during T cell receptor stimulation, mediated in part by the PD-1 receptor (9). The CheckMate 025 trial was an open label, randomized phase 3 study of patients with mRCC previously treated with antiangiogenic therapy which showed that second line treatment with nivolumab improved OS to 25.0 months compared to 19.6 months with the mTOR inhibitor everolimus (10). This led to the FDA approval of nivolumab in RCC and established it as the standard of care for mRCC in the second line setting. Several other checkpoint inhibitors including ipilimumab (anti-CTLA4; in combination with nivolumab), pembrolizumab (anti-PD1; in combination with axitinib), and avelumab (anti-PD-L1; in combination with axitinib) have been approved for the treatment of advanced RCC, with several others currently under investigation (11).

RCC has traditionally considered radioresistant (12). This is based on trials from the 1970's and 80's that utilized outdated radiation techniques and *in vitro* data describing methods of RCC resistance to radiation (13, 14). Newer data suggest that modern stereotactic ablative radiotherapy can bypass the resistance mechanism and can be an effective therapy both in the primary setting and in treating metastatic disease (14). More recently, there has been interest in interactions between radiation and immunotherapy. Here, we present a patient with mRCC with brain metastases who received checkpoint inhibitors and radiotherapy, with near complete response. We highlight relevant aspects of the case and discuss the current status of combining radiotherapy and immunotherapy in mRCC.

### Case Vignette

**Figure 1:**
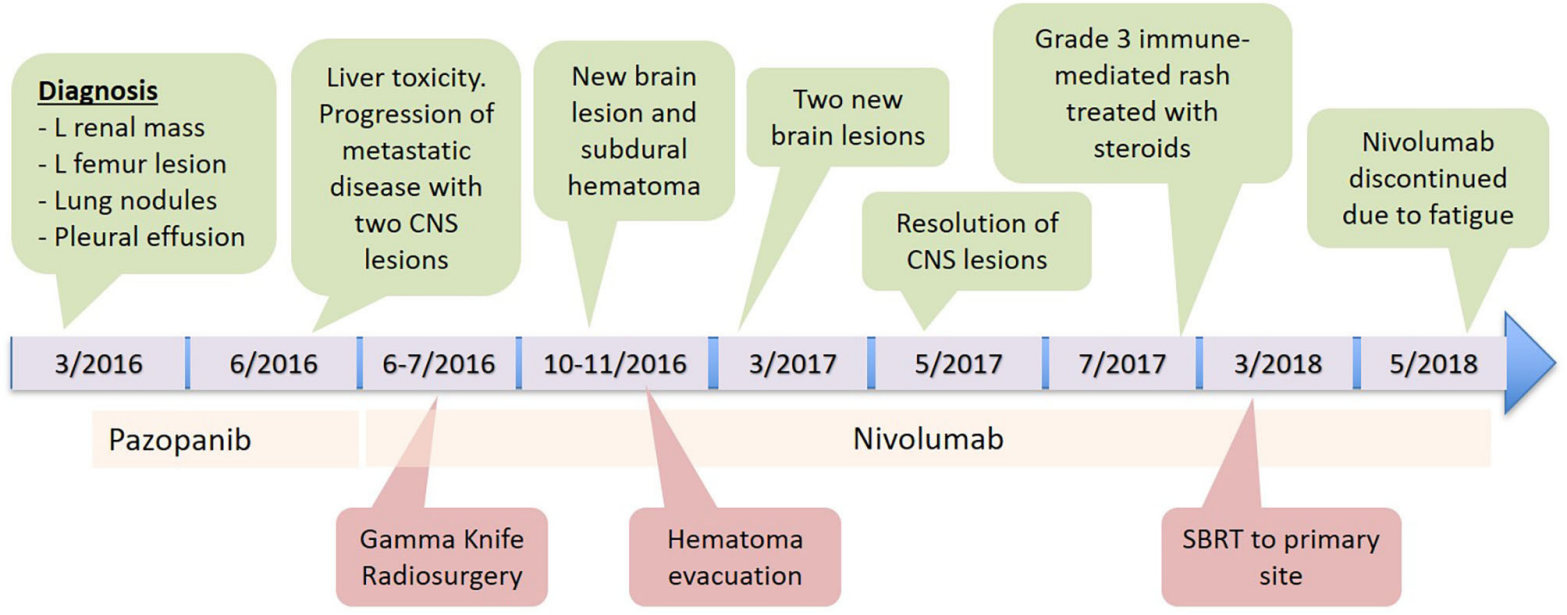
Timeline.

**Figure 2:**
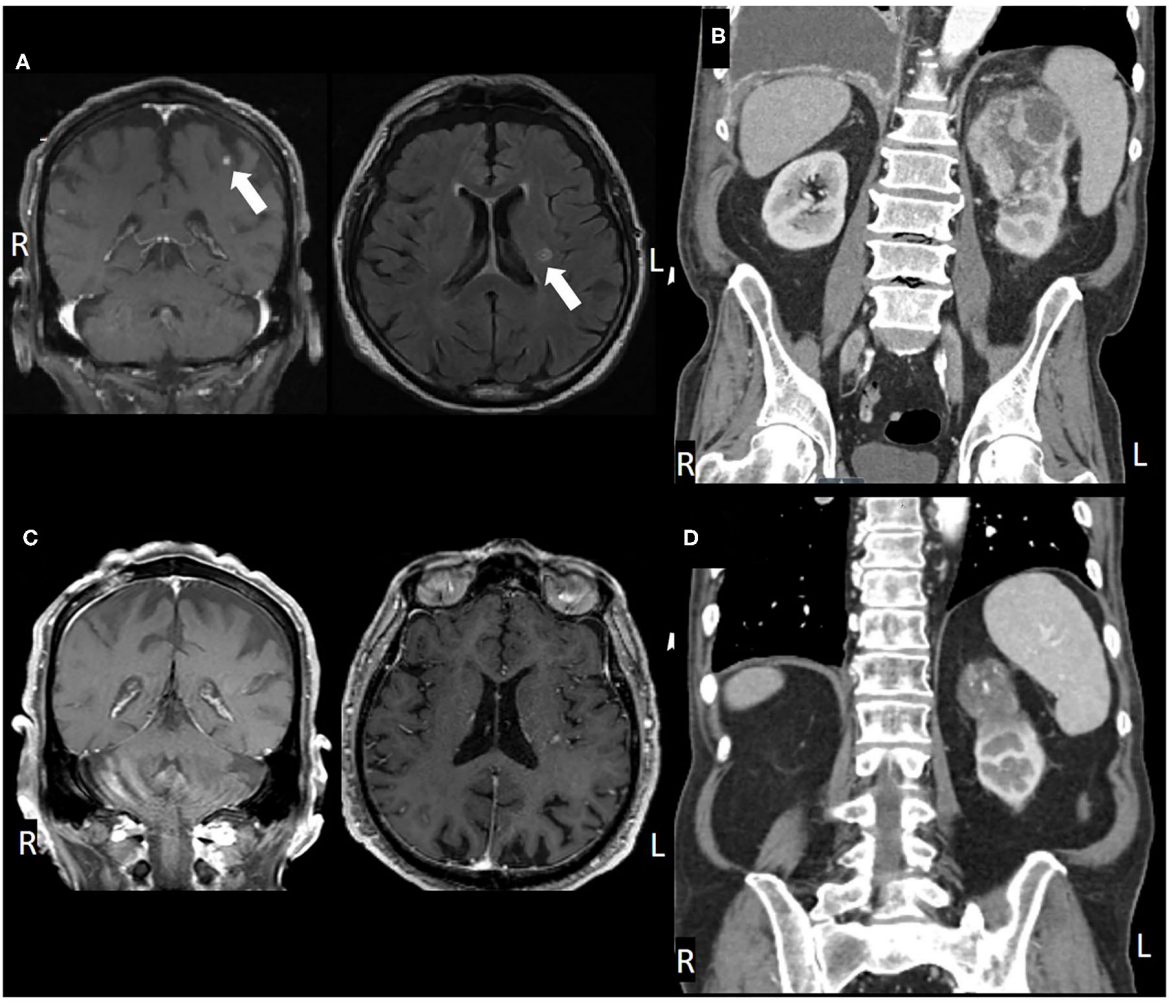
**(A)** Pre-treatment MRI, coronal, and axial views, **(B)** post-SRS and immunotherapy MRI, **(C)** pre-treatment CT, coronal view, and **(D)** post-treatment CT.

An 83-year-old male presented in March 2016 with progressive shortness of breath (Figure 1). CT angiogram (CTA) revealed multiple bilateral lung nodules, pleural effusion, and a large left kidney mass measuring 9.1 × 7.5 cm concerning for malignancy. Biopsy of a right pleural based mass demonstrated RCC, clear cell histology. Bone scan and plain-radiograph showed a single site of bone involvement, with a lytic lesion measuring 3.4 × 2.3 cm in the left femoral diaphysis. The patient was started on systemic therapy with pazopanib until he developed liver toxicity 2 months later. Shortly following cessation of the medication in June 2016, he again complained of dyspnea and new right foot drop. Imaging showed progression of metastatic disease with bilateral increase in size of pulmonary nodules, mediastinal lymphadenopathy, an additional osseous lesion in the pelvis, and growth of the primary lesion (Figure 2B). MRI of the brain and spinal axis revealed no cord compression, but two brain metastases in the left pre-central gyrus and left corona radiata were identified (Figure 2A). Second line therapy with nivolumab was initiated at that time. The CNS lesions, as well as two new 2 mm brain lesions in the right frontal pole and cerebellum identified at the time of treatment, were treated with gamma knife radiosurgery 20 Gy to the 50% isodose line in July 2016, after initiation of nivolumab.

Four months later in October 2016, the patient had a fall and brain MRI revealed a new 5 mm left posterior parietal lesion and a small subdural hematoma. He was initially planned for further radiosurgery, however, he became symptomatic with right arm weakness, and repeat imaging revealed hematoma expansion causing mass effect on the right ventricle and midline shift, increased size of a left post-central gyrus lesion and improvement in the left posterior parietal lesion. The patient underwent surgical evacuation of the hematoma in November 2016 without local therapy for the metastases. All neurologic deficits resolved, and the patient was asymptomatic from his disease.

Brain imaging in March 2017 discovered two new metastatic lesions measuring 6 mm in the left temporal lobe and 3 mm in the right frontal lobe. Due to his excellent functional status, the patient declined further radiosurgery in favor of continued immunotherapy with close monitoring. His next brain MRI in May 2017 revealed complete resolution of the two lesions (Figure 2). Therapy with nivolumab was generally well-tolerated, however, the patient did develop a grade 3 immune-mediated rash (confirmed by biopsy) in July 2017 after ~1 year on nivolumab therapy. He was treated with a prolonged steroid course, with eventual complete resolution of his rash.

In January 2018, after extensive discussion regarding the potential risks and benefits, he was re-started on nivolumab. The patient demonstrated stable disease with interval decrease in the size of the primary lesion. However, in consultation with Radiation Oncology, the decision was made to administer SBRT to the primary site. He was treated to 40 Gy in five fractions in March 2018. Nivolumab was continued until May 2018 but held at that time due to fatigue. The patient completed a total of 34 cycles of nivolumab in May 2018 and has not received additional therapy since then. The patient's most recent imaging in May 2020 showed a stable 4.6 cm left renal lesion with stable small pulmonary lesions and no other evidence of disease (Figures 2C,D).

## Discussion

Patients with mRCC have benefited from the paradigm shift brought about with the use of immune checkpoint inhibitors. However, patients with brain metastases are often excluded from these trials unless they have stable or treated disease (10, 15, 16). A subgroup analysis of patients from the phase 3 Checkmate 025 trial who continued nivolumab after progression of disease showed median OS of 22.5 months, compared to 12.3 months in subjects who were not treated beyond progression of disease (17). Although not the focus of the analysis, this cohort included patients who progressed due to brain involvement. The French expanded access trial, GETUG-AFU 26 (Nivoren), is a prospective phase II trial evaluating the efficacy and safety of nivolumab in patients with mRCC. Those with brain metastases (with or without previous focal treatment, not requiring steroids; ECOG at least 2) were followed prospectively for intracranial response rate. Seventy six (10.4%) subjects had brain metastases, 39 had not received focal treatment (cohort A), 34 had prior focal treatment (cohort B), and 3 did not undergo treatment due to performance status. Median follow up was 23.6 (95% CI = 18.1–24.6) months and 20.2 (95% CI = 16.3–22.9) months and median duration of treatment was 4.9 (range: 0.5–24.2) months and 4.5 (range 0.5–22.3) months for cohort A and B, respectively. Median intracranial PFS was 2.7 months (95% CI = 2.3–4.6) in cohort A and 4.8 months (95% CI = 3.0–8.0) in cohort B. Prior focal brain therapy, as in cohort B, decreased the risk of intracranial progression at a hazard ratio of 0.49 (95% CI = 0.26–0.92). This study highlights the role of focal treatment of brain metastases and notes that only four patients in cohort A achieved objective intracranial response. Despite the favorable intracranial PFS and lower incidence of symptoms due to brain metastases among cohort B (32% in cohort B, 49% in cohort A, there was no significant difference in 12-month overall survival (66.7% [CI = 49.6–79.1) cohort A and 58.8% [CI = 40.6–73.2] cohort B) (18, 19). The Italian expanded-access study consisted of patients who were treated with Nivolumab for a median of 7.2 months and included 32 (8.2%) patients with brain metastases that did not require steroids or radiotherapy. The median PFS was 4.4 months (95% CI = 3.7–6.2), ORR was 23.1 and 36.2% of patients had progressive disease. Overall survival at 6 and 12 months was 87 and 66.8% respectively, with median OS not reached at the time of analysis. Treatment-related adverse events were similar between the brain-metastatic patients and the overall population, but grade 3–4 toxicities were more common among those with brain metastases (12 vs. 7%) (20). These trials suggest improved outcome for brain metastatic patients with acceptable toxicity profiles. Indeed, the checkpoint 025 trial reported greater adverse events for patients treated with everolimus than with nivolumab (88%, compared with 79%) (10).

RCC has historically been considered a radioresistant tumor and the role of radiation has been limited to the palliative setting (21). Radioresistance was demonstrated *in vitro* in a 1995 meta-analysis in which RCC was found to have the highest survival among 694 human cell lines after irradiation to a dose of 2 Gy (13). The mechanism of resistance was proposed in 2005 and highlighted the role of the HIF-1 pathway (22). A feature of RCC is its loss of function mutation/methylation in the von Hippel-Lindau (VHL) tumor suppressor gene. This results in high levels of HIF-1α expression and upregulation of VEGF and downstream proangiogenic factors that protect epithelial cells and induce radioresistance. Clinical data seemed to uphold this concept. Two prospective studies in the 1970s investigated neoadjuvant RT followed by nephrectomy vs. nephrectomy alone in the non-metastatic setting failed to improve in 5-year survival (23, 24). Studies of adjuvant RT by Kjaer et al. (25) and Finney (26) similarly failed to improve survival and in fact, the 1973 study was concerning for a significant number of patient deaths (19.6%), in part due to radiation-induced liver damage. In contrast, data from Haimovitz-Friedman et al. (27) indicated that fractions larger than 8 Gy overcame the HIF-1 mediated resistance mechanism. These higher doses activate the cell surface sphingomyelin pathway to produce ceramide and induce apoptosis, providing an alternative pathway of cell death. This was first supported in human data by a Phase I dose-escalation study by Greco et al. (28) and a retrospective analysis by Vogelbaum et al. (29) both demonstrating good locoregional control and a dose-response (14).

Results from the first prospective trial to investigate the combination of multi-site SBRT in combination with pembrolizumab was reported at the 2018 ASCO-SITC Clinical Immuno-Oncology Symposium. This is a Phase I clinical trial studying the safety of SBRT and pembrolizumab in adults with advanced solid tumors who have progressed on standard of care treatment. Patients with active metastatic CNS disease were excluded from the trial. Seventy-six subjects underwent SBRT to up to four sites followed by pembrolizumab within 7 days of the final radiation treatment. Most patients (94.5%) received SBRT to two sites. Of the 76 subjects studied, only 1 case of RCC was included. Median follow up was 5.5 months. Six grade 3 toxicities were observed, including three cases of pneumonitis, two cases of colitis, and one case of hepatic failure. The ORR was 13.2%, significantly greater in irradiated than non-irradiated lesions (mean tumor diameter change 21.7% vs. 1.7%). Abscopal effect, which is immune-mediated tumor response outside of the radiation field, was observed in 13.5% of patients and was defined as a 30% reduction in the aggregate sum of nonirradiated lesions. A similar reduction in any single nonirradiated site was observed in 26.9% of patients (30). Two phase II trials evaluating the combination of immunotherapy and SBRT in mRCC were presented at the 2020 ASCO Genitourinary Cancers Symposium. The NIVES trial recruited 69 immunotherapy-naive patients with disease progression following at least two lines of anti-angiogenic therapy. Patients received SBRT 7 days following the first infusion of nivolumab and were continued on immunotherapy until disease progression or toxicity. The most common sites of SBRT were lung (37.7%), lymph nodes (11.6%), and bone (11.6%). At median follow up of 15 months, ORR was 17.4%, CR 1.4%, and disease control rate of 58%. Progression-free survival was 4.1 months, median OS was 22.07 months, and 1-year PFS and OS rates were 32.6 and 73.4%, respectively. Grade 3–4 toxicities were observed in 24.6% of patients and none of these occurred within the radiation field (31). RADVAX RCC evaluated the combination of nivolumab and ipilimumab with SBRT, with most patients having previously undergone nephrectomy (68%). The most common site for SBRT was lung (56%). Twenty-five participants were recruited and underwent SBRT to 1–2 sites between the first and second cycles of nivolumab-ipilimumab. Median follow up was 24 months. ORR was 56%, median PFS 8.21 months, and 1-year PFS rate 36%. The median OS and duration of response had not been reached. Grade 3–4 toxicities were reported in 36% of subjects (32). These two trials differed not only in single vs. dual immunotherapy regimens, but also in the radiation regimen. The RADVAX trial treated with SBRT to a dose of 50 Gy in five fractions, while the NIVES trial treated to a dose of 10 Gy in three fractions. It is not clear whether the favorable results from the RADVAX trial are due to the combination of nivolumab and ipilimumab, or more optimal dosing of SBRT. An interim analysis of CheckMate 920 features safety and efficacy results for the cohort of patients with brain metastases. Subjects who were previously untreated, had asymptomatic brain metastases not requiring steroids or radiation, and had a Karnofsky performance score of at least 70% were treated with nivolumab (3 mg/kg) and ipilumumab (1 mg/kg) every 3 weeks for four doses followed by nivolumab 480 mg every 4 weeks until disease progression or unacceptable toxicity for up to 2 years. Twenty-eight patients were enrolled with a median follow up of 6.47 months and analyzed for the primary endpoint of high-grade immune-mediated adverse events and secondary endpoints of progression free survival and objective response rate, with exploratory endpoints of safety analysis and overall survival. ORR was 28.6% (95% CI = 13.2–48.7), median PFS 9 months (CI = 2.9 – not estimable), and median OS had not been reached. There were six cases of grade 3–4 immune-mediated adverse events within 100 days of the last dose of immunotherapy. These data suggest that there may be a beneficial effect of SBRT which was reflected in results from the RADVAX trial; however, long term follow up and randomized data are required.

Further study is warranted, and future trials are underway. The CYTOSHRINK trial is a Phase II randomized trial that will include patients with advanced RCC who decline or are unsuitable for cytoreductive nephrectomy. Subjects will be randomized 2:1 to received ipilimumab or nivolumab plus SBRT (30–40 Gy in five fractions) to the primary kidney lesion vs. immunotherapy alone. The primary endpoint is the hazard ratio for PFS, and secondary endpoints are safety, OS, ORR, and health-related quality of life (NCT04090710) (33). There are several more ongoing phase I/II trials testing the use of stereotactic radiotherapy in combination with immunotherapy: NCT02864615 (SBRT in mRCC treated with targeted or immunotherapy), NCT02599779 (pembrolizumab + SBRT in TKI-refractory mRCC), NCT02781506 (nivolumab + SAbR in mRCC) (14) whose results will provide more insight into the effectiveness of such regimens for patients with RCC.

The combination of immune checkpoint inhibitors and radiation therapy may offer a pathway for achieving durable clinical response through the abscopal effect. Physiologic mechanisms responsible for the abscopal effect have been proposed primarily utilizing non-small-cell-lung-cancer (NSCLC) and melanoma as models. Twyman-Saint Victor et al. (9) utilized a B16-F10 melanoma mouse model with bilateral flank tumors and treated them with radiation to one tumor, anti-CTLA4 antibodies, or both treatments concurrently. The response rate in the concurrent treatment arm was 17%, consistent with a human phase I clinical trial in patients with metastatic melanoma, as reported by the same group. The top predictor of resistance was the CD8^+^/T_reg_ ratio, which failed to increase in resistant tumors but did increase in sensitive tumors. The mechanism of resistance was not mediated by factors contributing to radiation resistance, but rather factors that blunt the expansion of CD8 T cells. Among these, the most prevalent upregulated genes in resistant tumors were PD-L1 and interferon-stimulated genes. Elimination of PD-L1 using CRISPR restored response to radiation and CTLA-4 blockade and increased survival from 0 to 60%. Elevated levels of PD-L1 have been found to contribute to T-cell exhaustion. The addition of PD-1 or PD-L1 blockade to radiation and anti-CTLA4 in the mouse models increased complete response rates to 80% and was strongly correlated with reversal of exhaustion of CD8 T cells as well as an increase in the CD8/T_reg_ ratio. Additionally, assessment of T-cell receptors (TCR) revealed that irradiated tumors displayed increased diversity of TCR clonotypes compared to unirradiated tumors. TCR clonotype expansion was demonstrated in humans with NSCLC in a prospective study of the combination of radiation and CTLA-4 blockade (34). The study included patients who progressed after at least one prior treatment. Forty-one percent of patients on this trial had pre-existing brain metastases controlled by surgery or radiation. Patients received radiation to one metastasis and were treated with concurrent ipilimumab. The objective response rate was 33% in evaluable patients, with two patients achieving complete response. In contrast to the melanoma mouse model, in this study of human NSCLC neither PD-1 expression in pretreatment tumor nor CD8 T cell infiltration was associated with response. In addition, there was no evidence that PD-1+ T cell exhaustion was a factor in response. Instead, EGFR mutation, which is associated with poor response to PD-1/PD-L1 blockade (35), was significantly higher in patients with progressive disease than those with stable disease or partial/complete response. Similar to the melanoma mouse model, peripheral blood TCR clonotype diversity was associated with response, and had the highest predictive value. Ongoing clinical trials studying the abscopal effect in RCC include NCT02334709 (SBRT + TKI in mRCC) and NCT03469713 (NIVES trial).

Here, we have presented a case of RCC with brain metastases treated successfully with radiation and immunotherapy. The patient continues to follow in clinic with stable imaging and remains off treatment for over 2 years. His survival currently exceeds expectations based on the available data and historical averages. While anecdotal, this case is impressive and shows long term control of brain metastatic RCC, as this patient has remained off treatment, suggesting a robust treatment-induced immune response. While limited in the tissue correlatives, given the patient refused subsequent biopsies, his clinical course compared to patients treated with immunotherapy alone perhaps suggests some contribution of radiation. As mentioned, additional formal clinical study of the abscopal effect in mRCC is ongoing. Future avenues for research may investigate the optimal sequencing of immunotherapy and SBRT in patients with mRCC, the appropriate combinations and duration of treatment with immunotherapeutic agents, and broader inclusion of patients with CNS disease.

## Data Availability Statement

The original contributions presented in the study are included in the article/supplementary material, further inquiries can be directed to the corresponding author/s.

## Ethics Statement

The studies involving human participants were reviewed and approved by IRB protocol #20141135 Institutional Review Board of Washington University School of Medicine, St. Louis, MO. The patients/participants provided their written informed consent to participate in this study. Written informed consent was obtained from the individual(s) for the publication of any potentially identifiable images or data included in this article.

## Author Contributions

ML drafted the manuscript. JO collected patient data and organized relevant radiology images. WS and DC produced radiology and histology figures. RP conceived of the idea, contributed to manuscript writing, and oversaw the project. JH and MD treated patients and provided radiation treatment data. All authors reviewed and approved the manuscript.

## Conflict of Interest

RP reports consulting or advisory role for EMD Serono, Bristol-Myers Squibb, Pfizer/EMD Serono, Sanofi, Jounce Therapeutics, Dendreon, Bayer, and Genomic Health; speakers' bureau for Dendreon, Merck, Genentech/Roche, AstraZeneca, Sanofi, and Genomic Health; travel, accommodations, expenses from Genentech/Roche, DAVA Oncology; and research funding from Janssen Oncology. The remaining authors declare that the research was conducted in the absence of any commercial or financial relationships that could be construed as a potential conflict of interest.
